# Application of Hydrophobic Alkylimidazoles in the Separation of Non-Ferrous Metal Ions across Plasticised Membranes—A Review

**DOI:** 10.3390/membranes10110331

**Published:** 2020-11-06

**Authors:** Malgorzata Ulewicz, Elzbieta Radzyminska-Lenarcik

**Affiliations:** 1 Faculty of Civil Engineering, Czestochowa University of Technology, Dabrowskiego 69 Street, PL 42-201 Czestochowa, Poland; 2 Faculty of Chemical Technology and Engineering, UTP University of Science and Technology, Seminaryjna 3, PL 85-326 Bydgoszcz, Poland; elaradz@utp.edu.pl

**Keywords:** polymer inclusion membrane, alkylimidazole, metal ions, separation process

## Abstract

Currently, a lot of attention is paid to polymer inclusion membranes (PIMs). Their particular advantages include effective support fixation, easy preparation, versatility, stability, good mechanical properties and good chemical resistance. The paper presents a review of the literature related to the applications of polymer inclusion membranes containing alkylimidazole derivatives as carriers in the processes of transporting ions of heavy and toxic metals, such as Zn(II), Cu(II), Cd(II), Co(II), Ni(II), and Mn(II). It has been proven that alkylimidazoles exhibit varying complex-forming properties towards metal ions, and that their properties (hydrophobic and alkaline) can be modified easily by changing the size of the alkyl group and its position in the imidazole ring, which allows obtaining efficiently working metal ion carriers. The stability of an imidazole derivative-metal ion complex determines the speed and selectivity of the process of transporting metal ions across polymer inclusion membranes. Also, the morphological structure of polymer inclusion membranes impacts the efficiency of the process involving the release and separation of metal ions.

## 1. Introduction

The application of liquid membranes to the separation of metal ions dates back to 1967 [1]. Liquid membrane (LM) systems are increasingly being studied by scientists in many fields, for example, analytical, inorganic and organic chemistry, chemical engineering, biotechnology and biomedical engineering and wastewater treatment [2]. They are also employed for the treatment of water and the recovery of both toxic and economically valuable metals from used materials [3,4,5,6]. LMs are used for gas separation, as well as for the removal of organic compounds and many others [2,7]. The literature reports clearly show the wide range of application possibilities of the polymer inclusion membranes in many areas of the economy, including recycling and recovery processes [8,9]. Liquid membranes have been proposed as an alternative to conventional solvent extraction. In this type of membranes, the processes of extraction and re-extraction occur simultaneously. A liquid membrane constitutes a distinct organic phase separating two different water phases [2]. Compared to solvent extraction used for separation [10,11,12], membrane processes are characterised by better utilisation of an ion carrier (extractant), and they also eliminate the environmental hazards which result from the use of solvents utilised in traditional extraction (solubility in water, toxicity) [13].

Among all types of liquid membranes, the highest stability is exhibited by polymer inclusion membranes (PIMs). They are produced by pouring a solution being a mixture of a proper polymer, plasticiser and ion carrier on a neutral substrate. This simple technique allows easy modification of the composition of the membrane, which is very important since it enables affecting the efficiency and selectivity of the metal ion separation process [14,15]. The transport rate across PIMs is affected by a variety of parameters, such as: the type of polymer matrix, the concentration of the carrier, the amount of the plasticiser, the thickness of the membrane or membrane surface morphology [1,10,13,14,15,16,17,18,19]. The selectivity and stability of the action of liquid polymer membranes depend largely on the physicochemical properties of the plasticiser, and in particular on the ion carrier [1,16,20,21,22,23,24]. To achieve efficient transport of metal ions, different types of carriers with compatible base polymers have been successfully used [5,25].

The paper presents information on poorly soluble in water alkylimidazoles which were used to separate non-ferrous metal ions. The parameters and phenomena determining the suitability of alkylimidazoles for the separation of heavy metals are also presented. This work will be very useful in the appropriate selection of the carrier (alkylimidazole derivative), as well as in explaining the influence of the parameters characterizing PIMs, which have a direct impact on the stability of membranes, and the efficiency of transport, and separation of metal ions. Among the metals tested, special attention should be paid to copper(II) and zinc(II) ions due to the possibility of their selective separation from various types of solutions. The recovery of copper and zinc from wastewater and post-production solutions is important both for economic and environmental (ecological) benefits.

## 2. Properties of Alkylimidazoles

**Figure 1:**
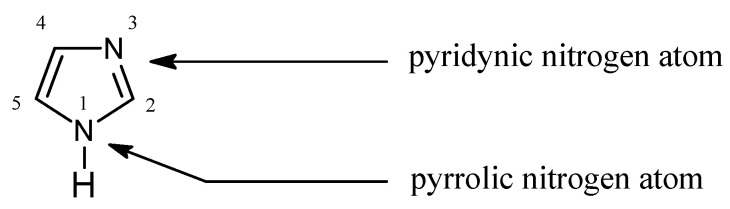
The imidazole molecule.

In Pearson’s theory [26] (HSAB), imidazole (Figure 1) is an intermediate base (pK_a_ = 7.14 [27]) forming stable complexes with metal ions, a feature which is used for their separation [28,29,30,31,32,33,34]:

The alkaline, hydrophobic and complexing properties of imidazole can be affected by the introduction of various substituents into the ring, including alkyl ones. Alkylimidazole derivatives constitute a group of stable bases, whose properties (hydrophobic and alkaline) can be modified by changing the size of the alkyl group and its position in the imidazole ring [35,36,37,38,39,40,41,42,43,44,45,46,47,48,49,50,51,52,53]. The ability to separate metal ions was tested with the use of the following homologous series of alkylimidazoles: 1-alkylimidazoles [35,36,37,38,39], 2-alkylimidazoles [51,52,53], 1,2-dialkylimidazoles [37,40,41,44,45,46,47,48,49] and 1,4-dialkylimidazoles [41,42,43,44,50]. It has been proven [54] that, for individual homologous series, the basicity increases linearly as the number of carbon atoms in alkyl groups becomes larger. The primary parameter deciding about the usefulness of alkylimidazoles for the separation of metals in the process of (solvent, membrane, etc.) extraction involves their basicity. 

Table 1 lists the values of dissociation constants (pK_a_) of the protonated forms (HL^+^) of the homologous series alkyl imidazole (L). The value of the constant term (the “b” constant) in linear equations may be a basis for comparing the basicity of homologous series of alkylimidazoles. The basicity of these homologues decreases in the following sequence: 1,2,4-trialkylimidazoles > 1,2-dialkylimidazoles > 2-alkylimidazoles > 1,4-dialkylimidazoles > 1-alkylimidazoles.

### 2.1. The 1-Alkylimidazole Moiety as a Carrier 

The placement of an alkyl substituent in the 1 position of imidazole causes a significant increase in the hydrophobic properties of the particle and a slight increase in its basicity (Table 1). 1-Alkylimidazoles are used as extractants of numerous metal ions, such as, e.g., Cu(II), Hg(II), Co(II), Pb(II) and Zn(II) [55,56]. Cuprey [55] and Shakers et al. [56] have suggested complex imidazole derivatives, having in their substituents in position 1,2 or 1,2,4,5 large alkyl groups with 6 to 40 carbon atoms, or cycloalkyl groups consisting of 5–12 carbon atoms.

**Figure 2:**
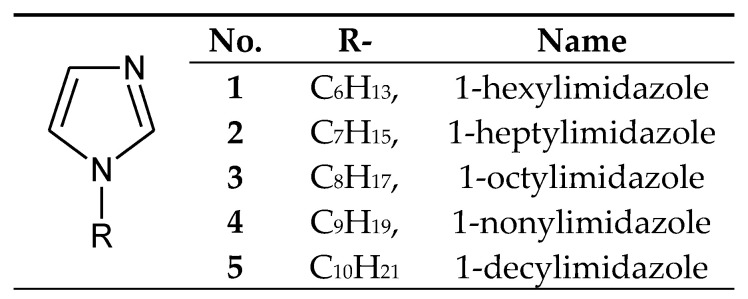
The 1-alkylimidazole molecule.

The processes of transport through the PIMs used 1-alkylimidazoles poorly soluble in water (Figure 2), having small alkyl substituents (from 6 to 10 carbon atoms).

1-Alkylimidazoles **1**–**5** (Figure 2) were used to separate Cu(II) from a Cu–Zn–Co–Ni mixture from nitrate [57,58] or chloride solutions [59], as well as Zn(II) from Zn–Co–Ni [60], Zn–Cd–Ni [61] and Zn–Mn mixtures [62]. Ajji and Ali [34] used 1-vinylimidazole to separate Cu(II) and Fe(III) ions in the process of transport across polymer membranes made of polyvinyl acetate.

### 2.2. The 1-Alkyl-2-Methylimidazole and 1-Alkyl-4-Methylimidazole as a Carriers

**Figure 3:**
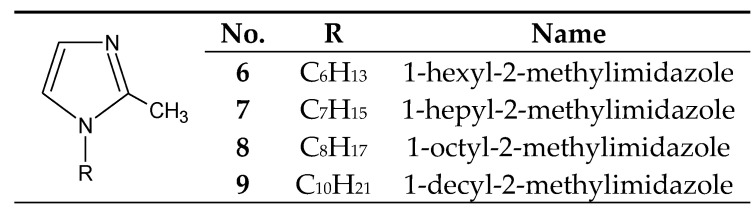
The 1-alkyl-2-metylimidazole molecule.

The introduction of even a small group (e.g., methyl) into the 2 or 4 positions of the imidazole ring highly increases the basicity of the molecule (Table 1), with a simultaneous increase in the hardness of these bases (the HSAB theory [26]) compared to imidazole and 1-alkylimidazoles. 1-Alkyl-2-methylimidazoles **6**–**9** (Figure 3) were used to separate Cu(II) from a Cu–Zn–Co–Ni [58,63,64,65] mixture, as well as Zn(II) from a Zn–Cd–Ni mixture [61].

**Figure 4:**
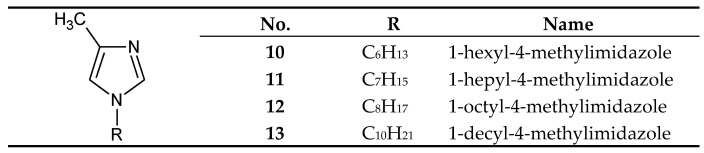
The 1-alkyl-4-metylimidazole molecule.

On the other hand, 1-alkyl-4-methylimidazoles **10**-**13** (Figure 4) were used to separate Cu(II) from a Cu–Zn–Co–Ni mixture [58] and Zn(II) from a Zn–Cd–Co–Ni [66,67] and a Zn–Cd–Ni mixture [61]. 1–Octyl–4–methylimidazole (**12**) was used in the final treatment of galvanic wastewater containing Zn(II) ions [68].

### 2.3. The 1-Alkyl-2,4-Dimethylimidazole as a Carrier

**Figure 5:**
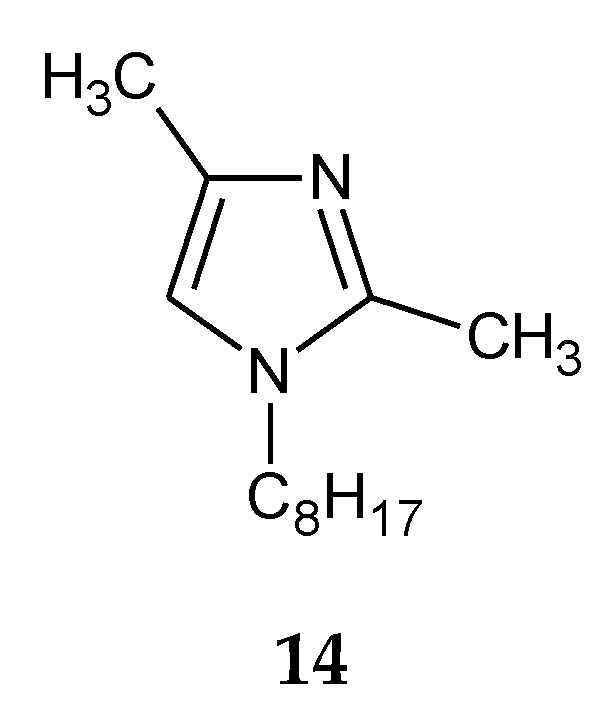
The 1-octyl-2,4-dimetylimidazole molecule.

**Figure 6:**
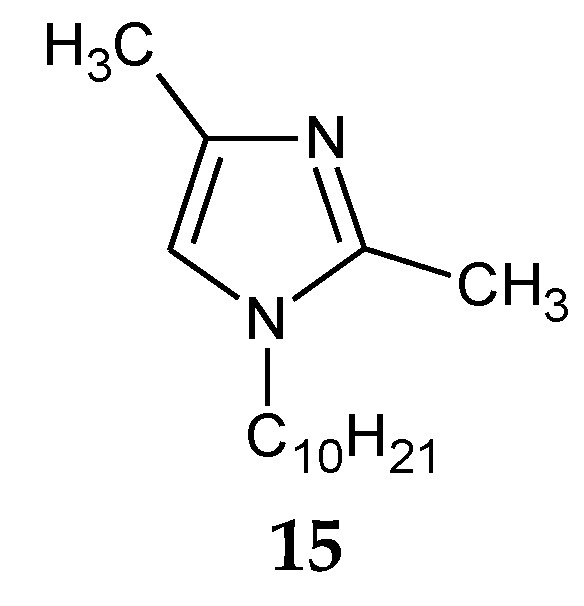
The 1-decyl-2,4-dimetylimidazole molecule.

The addition of two methyl groups into positions 2 and 4 of an imidazole ring highly increases the basicity of the molecule (Table 1). Among all homologous series of alkylimidazoles, 1-alkyl-2,4-dimethylimidazoles are the strongest bases. 1-Octyl-2,4-dimethylimidazole (**14**, Figure 5) was used to separate Zn(II) ions from a Zn–Cd–Ni mixture [61]. On the other hand, 1-decyl-2,4-dimethylimidazole (**15**, Figure 6) was used in the separation of Zn(II) from a Zn-Cd mixture [69].

## 3. Complexes of Alkylimidazoles

According to Bjerrum [70], in an aqueous solution, metal ions (M^z+^) having complex-forming properties gradually bind base particles (L) (Equation (1)):

[M(H_2_O)_N−n+1_L_n−1_]^z+^ + L ↔ [M(H_2_O)_N−n_L_n_]^z+^ + H_2_O (1)

in which N stands for the maximum coordination number of a metal ion.

**Figure 7:**
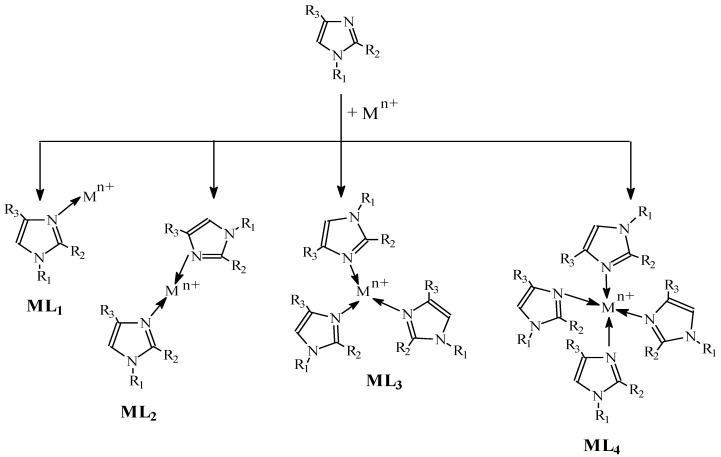
Complex formation.

This results in the establishment of equilibria for the gradual formation of single-core complexes (ML, ML_2_…..ML_N_). The formation of complexes with alkylimidazoles in a membrane proceeds according to the pattern presented in Figure 7.

Another factor deciding about the usefulness of alkylimidazoles for the separation of heavy metal ions involves the stability of their complexes with the ions of these metals. Table 2 lists the values of stability constants (log β_n_) of Co(II), Ni(II), Cu(II) and Zn(II) complexes with 1-alkylimidazoles.

Table 2 indicates that the stability of the complexes of Co(II), Ni(II), Cu(II) and Zn(II) ions increases in the following sequence: Co(II) < Zn(II) < Ni(II) < Cu(II). The values of stability constants of Cu(II) complexes with 1-alkylimidazoles do not depend on the length of the alkyl group [39], while the stability constants of Co(II) [35], Ni(II) [31] and Zn(II) [38] complexes increase linearly along with an increase in the length of the alkyl group (Table 2). Table 3 lists the values of stability constants (log β_n_) of Co(II), Cu(II) and Zn(II) complexes with selected 1-alkyl-2-methylimidazoles (**6**, **8**, **9**).

The data in Table 3 indicates that the stability of complexes of 1-alkyl-2-methylimidazoles with metal cations is lower by one order of magnitude compared to analogous compounds with 1-alkylimidazoles (Table 2), although 1-alkyl-2-methylimidazoles are harder bases and they should be bound more strongly with metal cations. Reduction in the stability of these complexes results from the so-called steric effect (steric hindrance) related to the presence of a substituent (e.g., a methyl) in direct vicinity of the donor nitrogen atom (Figure 1, N-3). It hinders the process of generating complexes. Table 4 lists the values of stability constants (log β_n_) of Co(II), Cu(II) and Zn(II) complexes with selected 1-alkyl-4-methylimidazoles (**10**, **12**, **13**).

Similar to the case of 1-alkyl-2-methylimidazoles, the steric effect of a methyl substituent in position 4 results in a reduction of the stability of complexes of 1-alkyl-4-methylimidazoles compared to analogous compounds with 1-alkylimidazoles. The influence of steric hindrance also depends on the complex forming properties of metal ions. It hinders the formation of octahedral or pseudo-octahedral complexes. It does not hinder the generation of complexes with the shape of a tetrahedron.

Table 4 indicates that the stability of the complexes of Co(II), Ni(II), Cu(II), Zn(II) and Cd(II) ions increases in the following sequence: Ni(II) < Cd(II), Co(II) < Zn(II) < Cu(II). In aqueous solutions, cations of Co(II), Ni(II), Cu(II), Zn(II), and Cd(II) exist in the form of octahedral aqua complexes [M(H_2_O)_6_]^2+^ [72]. In the case of Co(II), Zn(II) and Cd(II) ions, their octahedral aqueous complexes tend to change the coordinate number (c.n.) from 6 to 4, and at the same time change the symmetry of their coordination sphere into a square flat or deformed tetrahedron, in depending on the structure of their d-electron layer, which can be described in Equations (2):

[M(H_2_O)_6−n+1_L_n−1_]^2+^ + L ↔ [M(H_2_O)_4−n_L_n_]^2+^ + 3H_2_O [M(H_2_O)_6−n_L_n_]^2+^ ↔ [M(H_2_O)_4−n_L_n_]^2+^ + 2H_2_O (2)

The steric effect of the substituent in position 2 or 4 decreases the stability constants of octahedral complexes of all the studied metals, although it does not hinder the formation of tetrahedral species [37,40,41,42,43,44,45,46,47,51,71]. The Ni(II) ions form mostly 6-coordination complexes, because they have a rigid octahedral structure which is hard to deform. The complexes of Ni(II) both with 1-alkyl-2-methylimidazoles (Table 3) and with 1-alkyl-4-methylimidazoles (Table 4) have the lowest stability constants compared to 1-alkylimidazoles. The steric effect (Table 3 and Table 4) has a much lower impact on the stability of Cu(II) complexes due to the plasticity of its coordination sphere [73].

Table 5 lists the values of stability constants (log β_n_) of Co(II), Cu(II) and Zn(II) complexes with selected 1-alkyl-2,4-dimethylimidazoles **14**, **15**. In the case of 1-octyl-2,4-dimethylimidazole (**14**), the stability constants of Zn(II), Cd(II) and Ni(II) complexes increase in the following order: Zn(II) > Cd(II) > Ni(II) (Table 5); for 1-decyl-2,4-dimethylimidazole (**15**), complexes with Zn(II) are more stable than those with Ni(II), except for the first formed complex (ML). The steric effect caused by the presence of methyl substituents in positions 2 and 4 causes the values of stability constants for the complexes of metals (Table 5) with 1-alkyl-2,4-dimethylimidazoles to be lower compared to 1,2- and 1,4-dialkylimidazoles. 

## 4. Transport of Complexes across PIMs

The ionic radii and hydration energy of the cations of d-electron metals (heavy metals) are very similar. For Zn(II), Cu(II), Co(II) and Ni(II), ionic radii amount to 74, 73, 74.4, 69 pm [74], respectively, with hydration energies of 2940, 3000, 2920, 3000 kJ/mol [74]. Therefore, the separation and recovery of these cations from aqueous solutions (including wastewater) is difficult. However, when transferring metal ions into complexes with ligands other than water, their properties can be diversified and this can be used for their separation, e.g., by using differences in the transport of the formed complexes through polymer inclusion membranes (PIMs).

The process of transport involves the transfer of a complex generated in a reaction of a carrier (L) with a metal ion (M) (Equation (3)), the charge of the ion being compensated by anions present in the solution (X):

[M(H_2_O)_6_]^2+^ + nL + 2X^−^ ↔ [M(H_2_O)_6−n_L_n_]X_2_ + (n+2)H_2_O (3)

**Figure 8:**
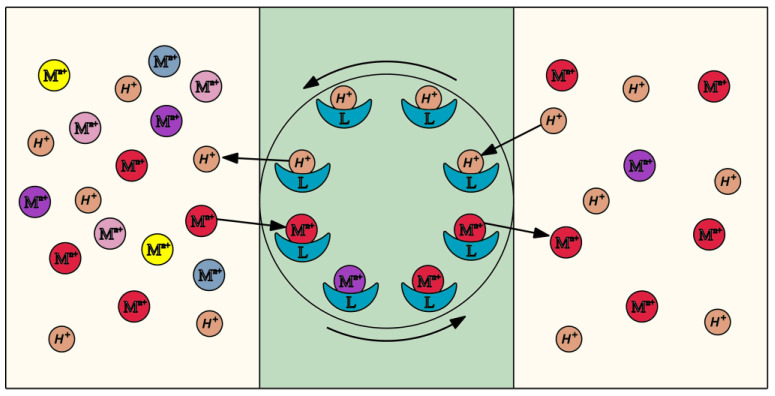
Transport of metal ions through the membrane: a layout.

This results in the generation of a hydrophobic complex which diffuses across the membrane (Figure 8). The metal ion is released into the receiving phase and the carrier undergoes further diffusion across the membrane to the supplying phase. This process continues until the moment of reaching a complete chemical equilibrium of the system. The use of a carrier with an alkaline nature causes countertransport, which is accompanied by the transport of hydrogen ions in an opposite direction (Figure 8). The transfer mechanism remains in compliance with the mechanism specified in the literature [75,76,77].

Due to their ability to form complexes with numerous metals, alkylimidazoles served the function of carriers in PIMs [57,58,59,60,61,62,63,64,65,66,67,68,69] based on CTA. In these membranes, *o*-nitrophenyl pentyl ether (*o*-NPPE) [57,58,59,60,61,62,63,64,65,66,67,69] or *o*-nitrophenyl octyl ether (*o*-NPOE) [68] were used as plasticisers. The amount of a plasticiser in the membrane was approximately 3–6%. The speed of transport of metal ions depends on the amount of the carrier (alkylimidazole) and increases along with an increase in its concentration [58,63]. For economic reasons, a carrier concentration of 1 mol/dm^3^ calculated for 1 g of CTA was considered as optimal [57,58,59,60,61,62,63,64,65,66,67,68,69]. Parameters defined by Danesi [78], characterising transport across membranes, such as: initial fluxes (J_0_), selectivity factors (S_M1/M2_) and recovery factors (RF), were compared in order to determine the efficiency of PMIs doped with alkylimidazole in the separation of copper and zinc ions from their mixtures with the ions of non-ferrous metals (Cu–Zn–Co–Ni–Cd).

## 5. Separation of Copper(II) 

Copper(II) ions are the best transported from equimolar 4-component mixtures (Cu–Zn–Cd–Ni) across PIMs, which consist of cellulose triacetate (CTA) as polymeric support, *o*-nitrophenyl pentyl ether (*o*-NPPE) as plasticiser and alkylimidazole (Figure 2, Figure 3 and Figure 4) as ion carrier (Table 6) [57,58,59,63,64,65].

As indicated by the data in Table 6, in the case of 1-alkylimidazoles as carriers, the initial fluxes of Cu(II) ions have the highest values, both in nitrate [57,58,63,64,65] and in chloride solutions [59]. In a temperature of 20 °C, with pH of the supplying phase amounting to 5.5 [63], 6.0 [57,59,64,65] and 6.8 [58], regardless of the length of the alkyl chain in position 1 in a carrier molecule (**1**–**9**, **11**), the ions are transported in the following sequence: Cu(II) > Zn(II) > Co(II) > Ni(II). The highest initial flux values for the transport of Cu(II) were observed for 1-decylimidazole (7.03 μmol/m^2^s) [57]. 

In the case of alkylimidazoles with the methyl group as a substituent in position 2 or 4, the values of initial fluxes are lower than for 1-alkylimidazoles and comparable, except for 1-hexyl-2-methylimidazole (**6**), for which the initial flux value for the transport of Cu(II) was the lowest [63,64]. A drop in the values of initial fluxes is related to the steric effect of the methyl substituent (Me) in the vicinity of the donor nitrogen atom, which hinders the formation of complexes. 

Table 7 lists the values of copper(II) separation coefficients compared to Zn(II), Co(II) and Ni(II) from equimolar 4-component Cu–Zn–Co–Ni solutions after a 24-h transport process. 

The results in Table 7 show that Cu(II) can be separated very efficiently from other heavy and transition metal cations like Zn(II), Co(II) and Ni(II) [57,58,59,63], both in nitrate [57,58,63] and chloride [59] solutions. In the case of 1-alkylimidazoles **1**–**5**, the selectivity of transport of Cu(II) ions compared to the remaining metals decreases along with an increase in the hydrophobicity of the carrier molecule, since there is an increase in the speed of transport of Zn(II), Co(II) and Ni(II) ions [57,58,59]. The highest values of Cu/Zn, Cu/Co and Cu/Ni separation coefficients were achieved for 1-hexylimidazole (**1**). They amount to 4.3, 39.7 and 46.9, respectively [57]. The separation coefficients of all ions are higher in nitrate solutions [57] than in chloride solutions [59] (Table 7). In the case of 1-alkyl-2-methylimidazoles (**6**–**9**) [58,63] and 1-heptyl-4-methylimidazole (**11**) [58], Cu(II)/M(II) separation coefficients are lower than for 1-alkylimidazoles, except for Cu/Ni, when 1-hexyl-2-methylimidazole (**6**) is a carrier in the membrane (S_Cu(II)/Ni(II)_ = 59.1 [63]).

Table 8 contains the values of Cu(II) recovery factors in transport across PIMs doped with alkylimidazoles. The data in Table 8 indicate that copper recovery factors after a 24-h process of transport using alkyl imidazole derivatives as carriers are high, which proves the efficiency of alkylimidazoles in the process of separation of Cu(II) ions. The highest Cu(II) recovery factors were achieved when using 1-alkylimidazoles **1**, **2** [57].

## 6. Separation of Zinc(II) 

Zinc(II) ions are best transported across PIMs which consist of cellulose triacetate (CTA) as a polymeric support, o-nitrophenyl pentyl ether (*o*-NPPE) as a plasticiser and alkylimidazole (Figure 2, Figure 3 and Figure 4) as an ion carrier (Table 9) from equimolar 2-component (Zn–Cd [69] or Zn–Mn [62]), 3-component (Zn–Cd–Ni [60,61] or Zn–Co–Ni [60]) and 4-component (Zn–Cd–Co–Ni) mixtures [66,67]. Zinc can also be successfully recovered from galvanic wastewater containing Zn–Ni [68]. In each case, Zn(II) ions are best transported across PIMs doped with alkylimidazoles, in chloride, sulphate, as well as nitrate solutions [60,61,62,66,67,68,69].

The data in Table 9 indicates that the highest initial fluxes of Zn(II) ions were achieved when using 1-alkyl-2,4-dimethylimidazoles **14**, **15** as carriers (25.44 µmol·m^−2^s^−1^), as well as in the case of PIMs doped with 1-octyl-imidazole (**3**) (10.76 µmol·m^−2^s^−1^). For a 4-component Zn-Cd-Co-Ni solution, initial fluxes for the transport of metal ions across PIMs containing 1-hexyl-4-methylimidazole (**10**) or 1-decyl-4-methylimidazole (**13**) decrease in the following order: Zn(II) > Cd(II) > Co(II) > Ni(II) [66,67]. For a 3-component Zn–Cd–Ni solution [61], for PIMs doped with 1-octyl-imidazole (**3**), 1-octyl-2-methylimidazole (**8**), 1-octyl-4-methylimidazole (**12**) or 1-octyl-2,4-dimethylimidazole (**14**), the initial fluxes of metal ions decrease in the following order: Zn(II) > Cd(II) > Ni(II) [61]. For a Zn–Co–Ni mixture, the initial fluxes of metal transport for PIMs doped with 1-alkyl-imidazole (**1**–**5**) decrease in the following order: Zn(II) > Co(II) > Ni(II) [60].

Zinc easily forms 4-coordinate complexes with carriers whose molecules are affected by steric hindrance, following the equation:

[Zn(H_2_O)_6_]^2+^ + nL ↔ [Zn(H_2_O)_4−n_L_n_]^2+^ + (2+n)H_2_O (4)

wherein L stands for a 1-alkyl-4-methylimidazole, 1-alkyl-2-methylimidazole or 1-alkyl-2,4-dimethylimidazole molecule.

Co(II) and Cd(II) ions are also among those which, apart from 6-coordinate complexes, can form 4-coordinate complexes too:

[CoL_4_(H_2_O)_2_]^2+^ ↔ [CoL_4_]^2+^ + 2H_2_O (5)

[CdL_4_(H_2_O)_2_]^2+^ ↔ [CdL_4_]^2+^ + 2H_2_O (6)

wherein 6-coordinate complexes cannot be generated until the third or fourth stage of complexation (Table 3, Table 4 and Table 5), with a higher concentration of the carrier [37,43,47,66,67,69]. Ni(II) ions have a rigid coordination sphere; they form 6-coordinate complexes and they are practically not transported across this type of membranes (very low initial fluxes). This is advantageous from the standpoint of separation of metals, since these ions practically remain in the supplying phase [60,61,66,67,68].

Table 10, Table 11 and Table 12 list the values of zinc(II) separation coefficients compared to non-ferrous metal ions from equimolar 4-component Zn–Co–Cd–Ni (Table 10), 3-component Zn–Co–Ni (Table 11) and 2-component Zn–Cd, Zn–Co, Zn–Mn, Zn–Ni (Table 12) solutions after a 24-h process of transport across PIMs doped with alkylimidazoles.

Zn(II) separation coefficients compared to Cd(II), Co(II) and Ni(II) ions in 4-component Zn–Cd–Co–Ni mixtures (Table 10) are higher when using 1-hexyl-4-methylimidazole (**10**) [67] as a carrier in PIMs, except for S_Zn(II)/Co(II)_, for which this factor is slightly higher for **13** [66].

The ability to form tetrahedral complexes of Zn(II), Cd(II) and Co(II) is not advantageous in the separation of these ions, especially in the case of zinc and cadmium, which are in the same group of the periodic table and their properties are similar. For both carriers (**10** and **13**), Zn/Cd separation coefficients are comparable. For both carriers used in PIMs (**10**, **13**), the resulting Zn/Ni separation coefficients were high, with them being higher in the case of doped PIMs (**10**).

In the case of 3-component Zn–Co–Ni mixtures (Table 11), for 1-alkylimidazoles **1**–**5** as carriers, Zn/Co and Zn/Ni separation coefficients decrease along with an increase in the length of the alkyl substituent in position 1, and they are the highest in the case of 1-hexylimidazole (**1**) [60]. The separation coefficient can be increased using **10** as a carrier in the membranes, especially compared to Ni(II) ions, for which the Zn/Ni separation coefficient increases slightly more than two times.

Zinc separation coefficients from nitrate, sulphate and chloride solutions compared to manganese, cadmium, cobalt and nickel in 2-component mixtures are listed in Table 12.

1-Alkylimidazoles **1**–**5** were applied as carriers in PIMs used to separate a mixture of Zn–Mn ions in sulphate solutions [62]. The values of separation coefficients drop along with an increase in the length of the alkyl substituent in position 1 and they are the highest in the case of 1-hexylimidazole (**1**) (Table 12). In the case of a Zn–Cd mixture, it was not until 1-decyl-2,4-dimethylimidazole (**15**) was used as a carrier in PIMs [69] that a high separation coefficient could be achieved for these very similar metals (24.7). This was enabled by the structure of the carrier molecule (**15**), which causes enormous steric hindrance and impedes the formation of Cd(II) complexes. High Zn/Co and Zn/Ni separation coefficients were achieved using 1-decyl-4methylimidazole (**13**) as a carrier in PIMs, amounting to 27.3 and 22.4, respectively [66].

The percentage of zinc recovery from 2-, 3- and 4-component mixtures after a single 24-h process of transport across PIMs doped with 1-alkylimidazoles **1**-**5**, 1-alkyl-2-methylimidazoles **8**, **9**, 1-alkyl-4-methylimidazoles **12**, **13** and 1-alkyl-2,4-dimethylimidazoles **14**, **15** is presented in Table 13.

When using alkylimidazoles as carriers in PIMs, high zinc recovery values were achieved for all the tested mixtures (Table 13). Depending on alkylimidazole used as a carrier, recovery factors after a 24-h transport range from 76% to 97%. The higher the hydrophobicity of 1-alkylimidazoles **1**–**5**, the higher the degree of zinc separation, both in 2-component (Zn–Co [60], Zn–Mn [62]) and 3-component (Zn–Co–Ni [60]) solutions. A higher amount of zinc from 3-component Zn–Cd–Ni solutions is transported across membranes containing alkylimidazole as a carrier, whose molecule causes a steric effect in the form of complexes transported across the membrane [61]. The recovery factor of Zn(II) ions from a 4-component Zn–Cd–Co–Ni solution amounts to 96.9% [66].

## 7. Characteristics of Membranes

Although Kim et al. [79] demonstrated that the transport of Cs(I) ions across membranes with a calix[4]-crown-6-derivative did not depend on the structure of the membrane, most authors think [80,81,82,83,84,85,86,87,88,89,90,91] that the microstructure of the membrane surface is one of the important aspects influencing the transport of metal ions [87,88,89,90,91]. To this date, only a few studies [83,84,85,86,87,88,89] have been published involving membrane morphology. In the case of PIMs, created by the pouring of a polymer, plasticiser and carrier solution, the structure of the formed membrane differs depending on the types and concentrations of substances. The surface microstructure of the membrane material was examined in order to determine its basic parameters, such as the distribution of the support in the polymer matrix or surface porosity and roughness. Among the various surface testing techniques from papers [57,58,61,63,64,65,67,68,69] describing the application of imidazole alkyl derivatives as carriers in PIMs, the ones which were used included scanning electron microscopy (SEM) and atomic force microscopy (AFM).

### 7.1. SEM Studies of PIMs Doped Alkylimidazoles

**Figure 9:**
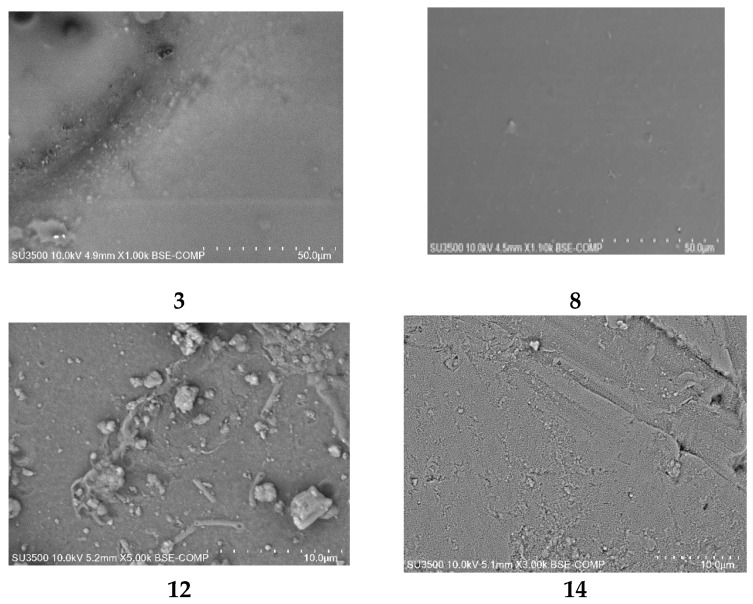
SEM-views of PIMs with 1-octylimidazole (**3**), 1-octyl-2-methylimidazole (**8**), 1-octyl-4-methylimidazole (**12**), and 1-octyl-2,4-dimethylimidazole (**14**) [61].

PIMs doped with alkylimidazole were characterised using the scanning electron microscopy (SEM) technique. A sample SEM image of PIMs doped with alkylimidazoles **3**, **8**, **12**, **14** is shown in Figure 9 [61]. 

The SEM photomicrographs show that all PIMs had dense and homogeneous structures, and also the roughness of the film could be observed. Carriers (alkylimidazole molecules) could crystallise in the membrane, causing its roughness and porosity [61]. PIMs containing alkylimidazoles **3**, **8**, **12**, **14** are dense and homogeneous.

### 7.2. AFM Studies of PIMs Doped Alkylimidazoles

**Figure 10:**
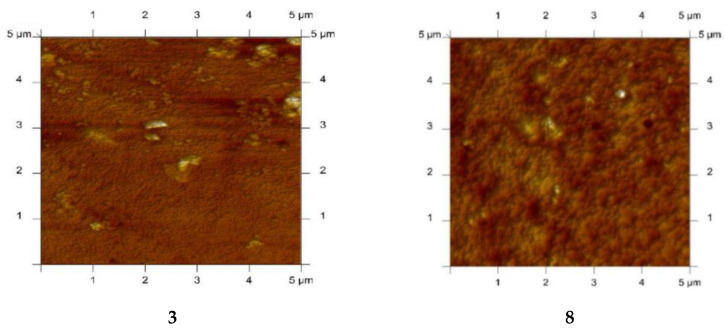

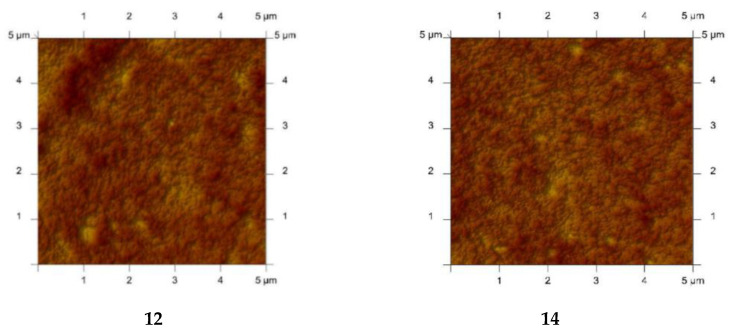
2D-view atomic force micrographs of PIMs with 1-octylimidazole (**3**), 1-octyl-2-methylimidazole (**8**), 1-octyl-4-methylimidazole (**12**), and 1-octyl-2,4-dimethylimidazole (**14**), [61].

PIMs with alkylimidazole were characterised using the atomic force microscopy (AFM) technique. A sample AFM image of PIMs with alkylimidazoles **3**, **8**, **12**, **14** in two-dimensional forms is shown in Figure 10 [61]. 

The AFM images in Figure 10 indicate that the distribution of carriers (1-octylimidazole (**3**), 1-octyl-2-methylimidazole (**8**), 1-octyl-4-methylimidazole (**12**) and 1-octyl-2,4-dimethylimidazole (**14**)) in the investigated membranes after the evaporation of the solvent is homogeneous throughout the entire surface. Similar images were obtained using **1**, **6**, **9**, **10**, **11**, **15** as carriers [57,58,63,64,65,67,68,69]. The membranes have well-defined pores that appear as small dark areas in AFM images.

Parameters characterising the surface (roughness (R_q_), porosity (ε)) were determined for PIMs with alkylimidazoles based on the membrane surface analysis (NanoScope software, v. 5.12). Also, their tortuosity (τ) was calculated from the Wolf and Strieder equation [90]. These parameters are listed in Table 14.

Based on the data in Table 14, it appears that the effective pore size in CTA-o-NPPE-alkylimidazole membranes varies from 0.050 to 0.065 µm and depends on the carrier used. The tortuosity and roughness of these membranes vary from 2.32 to 2.85 and from 2.2 to 6.7 nm, respectively.

The roughness of CTA-*o*-NPPE-alkylimidazole membranes is comparable to that found in PIMs with an imidazole derivative of azothiacrown ethers (3.3–5.3 nm) [91]. Roughness depends on the carrier concentration in the membranes. For PIMs doped with carrier **1** at a concentration of 1 mol/dm^3^, the roughness was 3.9 nm, while for a concentration of 1.5 mol/dm^3^ it was slightly lower (3.3 nm) [57].

## 8. Thermal Stability of PIMs Doped Alkylimidazoles

PIMs with alkylimidazoles were also tested for their thermal stability [57,60,62,65,66,67,68]. The thermal stability of these membranes was determined along with the ranges of temperatures and thermal effects of their degradation. It was demonstrated [81,83,85] that the degradation of a CTA-made membrane occurs in two steps; the first one over a range of 292–320 °C (main step) and the other one over a range of 450–476 °C (the charring of products).

**Figure 11:**
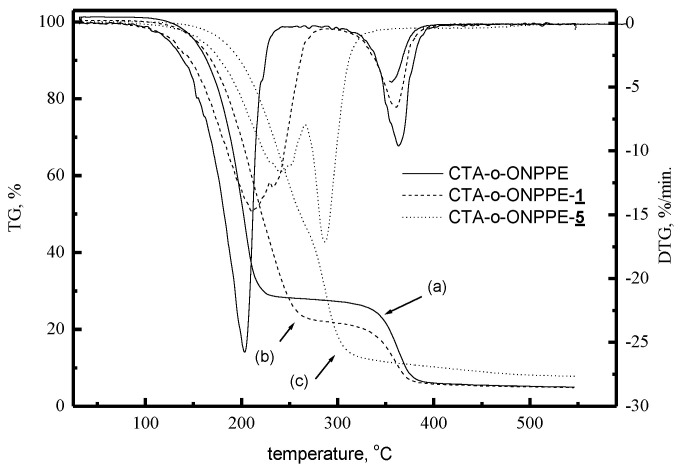
TG-DTA curves for membranes CTA-o-NPPE without carrier 1 (**a**), carrier 1 (**b**) and carrier 5 (**c**) [60].

Figure 11 shows sample thermograms of CTA-*o*-NPPE membranes with and without 1-hexylimidazole (**1**) and 1-decylimidazole (**5**). The degradation of CTA-o-NPPE membranes without a carrier occurs in two steps. In the first one, at 203.1 °C, a 73.5% loss in mass was recorded, and in the second one, at 363.3 °C, the loss amounted to 19.9% [60]. Also, the degradation of CTA-o-NPPE membranes with alkylimidazoles proceeds in two steps. The decomposition temperature ranges and the corresponding weight losses for CTA-*o*-NPPE membranes with alkylimidazoles are summarised in Table 15.

PIMs made of CTA-o-NPPE with alkylimidazoles show high thermal stability (up to approx. 200 °C) (the data in Table 15). For these membranes, the first step of degradation occurs at 211.3–251.3 °C, while the second one at 327–370 °C. The corresponding weight losses are within ranges of 61.3–80.57% and 5.12–18.90%, respectively.

## 9. Membrane Diffusion Coefficients of Non-Ferrous Metal Ions across PIMs with Alkylimidazoles

The calculated diffusion coefficients of non-ferrous metal ions across PIMs with alkylimidazoles are presented in Table 16 [59,61,63,67]. 

The normalised diffusion coefficient (D_0,n_) which considers morphological features inside a membrane (porosity, tortuosity) was calculated from the equation of Salazar-Alvarez et al. [92]. The values of diffusion coefficients range from 10^−12^ to 10^−8^ cm^2^/s and they show that the limiting step of the process involves the transfer of a metal complex across the membrane barrier. The values of normalised diffusion coefficients of M(II)-alkylimidazole complexes, from equimolar Cu(II)–Zn(II)–Co(II)–Ni(II), Zn(II)–Cd(II)–Ni(II), Zn(II)–Cd(II)–Co(II)–Ni(II) solutions, range from 10^−13^ to 10^−9^ cm^2^/s, and in every case they are the lowest for Ni(II) ions. Thus, the rate of transport of Cu(II), Zn(II), Co(II), Ni(II) ions across PIMs doped with alkylimidazole is determined by the diffusion rate of their M(II)-carrier complexes across the membrane.

## 10. Other Imidazole Derivatives in the Separation of Metal Ions

**Figure 12:**
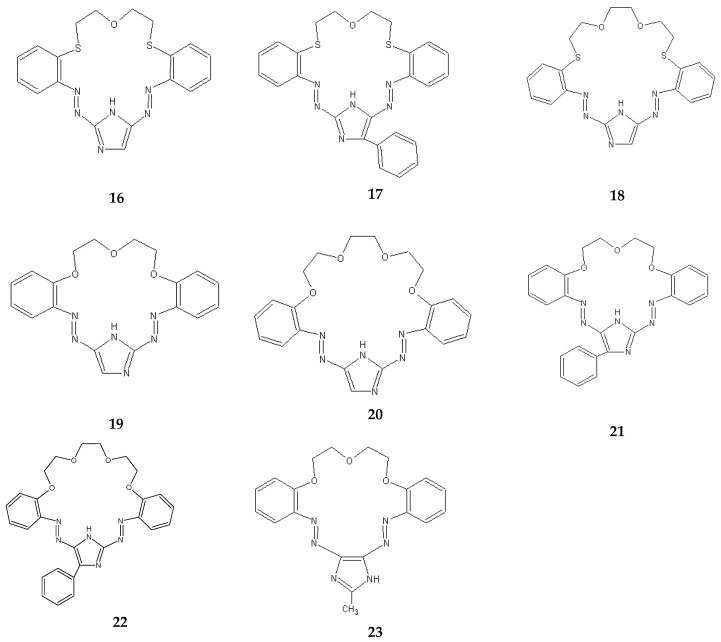
Imidazole derivatives of azo- and azothia-crown ethers used in the transport of metal ions across liquid membranes.

The separation of metal ions also used imidazole derivatives of crown ethers **16**–**23** containing two azo groups –N=N–, and in the case of carriers **16**–**18**, additionally sulphur atoms (instead of oxygen) (Figure 12). 

In the case of imidazole derivatives of azothiacrown ethers **16**–**18**, the highest values of Pb/Zn and Pb/Cd selectivity factors achieved for an imidazole derivative of an azothiacrown ether **17** amount to 19.5 and 105.4, and the selectivity of separation decreases in the following sequence: Pb(II) > Zn(II) > Cd(II). The use of the azothiacrown ether **17** allows the separation of over 90% of Pb(II) from the solution (after 48 h) [91]. On the other hand, for azocrown ethers **19**–**23**, the highest values of the selectivity factor for Pb/Zn and Pb/Cd ions achieved when using carriers **22** and **21** amount to 17.4 and 46.0, respectively. The use of the azocrown ether **21** allows over 96% separation of Pb(II) ions from the solution (after 24 h). In the process of transport across polymer membranes containing azocrown ethers **19**–**23**, the selectivity of separation decreases in the following sequence: Pb(II) > Zn(II) > Cd(II) [93,94]. The sequence of selectivity does not change along with an increase in the concentration of the carrier in the membrane within the range of concentration of the azocrown ether: 0.01–0.1 M. Also, a change in the concentration of hydrochloric acid in the receiving phase causes no change in the selectivity of separation of Zn(II), Cd(II) and Pb(II) ions from the solution in the process of transport across PIMs containing the derivatives of azocrown ethers. The use of a 0.5 M hydrochloric acid solution in the receiving phase causes a slight drop in the speed of transporting Zn(II), Cd(II) and Pb(II) ions across a polymer membrane, but the Pb/Cd and Pb/Zn selectivity factors are slightly increased, and for the azocrown ether **19** they amount to 9.5 and 45.7, respectively [93]. Since the adjustment of diameters of Pb(II), Cd(II) and Zn(II) cations for coordination number 6 to match the cavities of 18- and 21-member imidazole derivatives of azo- and azothiacrown ethers according to the CPK model decreases in the following sequence of cations: Pb(II) > Cd(II) > Zn(II), it can be concluded that in this case the rules coming from the HASB theory [26] probably outweigh the mismatch between the size of the cation and the cavity of the ligand. Polymer inclusion membranes containing the imidazole derivatives of azo- and azothiacrown ethers exhibit good stability of work. Initial flux for the transport of Zn(II), Cd(II) and Pb(II) ions across PIM membranes containing carrier **21** is stable for 240 h (four cycles of 48 h), with 288 h for carrier **17** (six cycles of 48 h) [91,94].

## 11. Conclusions

A membrane process using polymer inclusion membranes (PIMs) allows fulfilling two basic objectives, namely cleansing the solutions of metal cations, as well as separating their mixtures. The fulfilment of the first and second objective depends on proper selection of a carrier for metal ions and the conditions for carrying out the experiment. Alkylimidazole derivatives are efficient carriers of non-ferrous metal ions, such as Cu, Zn, Cd, Co, Ni and Mn. Their complex-forming can be easily differentiated, and differences in the processes of formation, structure and stability of their complexes with metal cations can be used for selective separation of metal ions from mixtures. Proper alkylimidazole derivatives can be selected for the purposes which are set when separating metal-bearing mixtures. 1-Alkylimidazoles allow reaching high values of recovery factors (RF), especially for Cu and Zn, but they do not guarantee high separation coefficients. The 2- or 4-substituted alkylimidazole derivatives allow achieving high separation coefficients, especially when separating Zn–Cd or Co–Ni mixtures. An increase in the hydrophobicity of alkylimidazole molecules decreases the selectivity of the process. As can be seen from the presented review, alkylimidazole derivatives can be appropriately selected depending on the effects we want to obtain by separating mixtures containing metals. Moreover, the imidazole derivatives are cheap ion carriers, which will have an impact on the cost-effectiveness of this technology when applied on a large scale.

The selectivity of transport across PIMs depends not just on the composition of the supplying phase and the composition of the receiving phase, but also on the composition and morphology of the membrane. The use of alkylimidazole derivatives PIMs will undoubtedly be of great interest to the separation or removal of heavy metals from aqueous solutions (including wastewater), for example to solve major environmental problems.

## Figures and Tables

**Table 1:** Changes in the basicity of alkylimidazoles depending on the position and number of alkyl groups according to [54], in aqueous solutions at 25 °C, ionic strength 0.5 mol/dm^3^ (KNO_3_).

Homologous Series	Linear Equation for Basicity pK_a_ = an + b
1-alkylimidazole	pK_a_ = 0.0222 n + 7.165
1,2-dialkylimidazole	pK_a_ = 0.0432 n + 8.01
1,2,4-trialkylimidazole	pK_a_ = 0.0503 n + 8.46
1,4-dialkylimidazole	pK_a_ = 0.0279 n + 7.79
2-alkylimidazole	pK_a_ = 0.0349 n + 7.87

n—is the sum of carbon atoms in the alkyl substituents.

**Table 2:** The stability constants (log β_n_) of Co(II), Ni(II), Cu(II), and Zn(II) complexes with 1-alkylimidazoles in aqueous solutions at 25 °C, ionic strength 0.5 mol/dm^3^ (KNO_3_).

log β_n_	Co(II) [35]	Ni(II) [31]	Cu(II) [39]	Zn(II) [38]
log β_1_	y = 0.302x + 1.653	y = 0.161x + 2.631	4.15	y = 0.229x + 1.986
log β_2_	y = 0.342x + 2.592	y = 0.164x + 4.790	7.57	y = 0.229x + 4.500
log β_3_	y = 0.377x + 3.881	y = 0.164x + 6.233	-	y = 0.229x + 5.700
log β_4_	y = 0.434x + 4.780	y = 0.166x + 5.653	-	-

x—the number of carbon atoms in the 1-alkyl substituent.

**Table 3:** The stability constants (log β_n_) of Co(II) [41], Cu(II) [40], and Zn(II) [40] complexes with 1-alkyl-2-methylimidazoles in aqueous solutions at 25 °C, ionic strength 0.5 mol/dm^3^ (KNO_3_).

Carrier	Metal Ion	log β_1_	log β_2_	log β_3_	log β_4_
1-hexyl-2-methylimidazole (**6**)	Co(II)	1.96	2.18	3.02	5.61
	Cu(II)	3.52	6.63	8.98	-
	Zn(II)	3.48	5.80	8.30	10.10
1-octyl-2-methylimidazole (**8**)	Co(II)	1.94	2.14	3.06	5.70
	Cu(II)	3.53	6.65	9.65	-
	Zn(II)	4.45	6.80	9.10	-
1-decyl-2-methylimidazole (**9**)	Co(II)	1.95	2.21	3.16	5.76
	Cu(II)	3.54	6.68	9.44	-
	Zn(II)	5.10	7.75	9.90	-

**Table 4:** The stability constants (log β_n_) of Co(II), Cu(II), Zn(II), Cd(II), and Ni(II) complexes with 1-alkyl-4-methylimidazoles in aqueous solutions at 25 °C, ionic strength 0.5 mol/dm^3^ (KNO_3_).

Carrier	Metal Ion	log β_1_	log β_2_	log β_3_	log β_4_
1-hexyl-4-methylimidazole (**10**)	Co(II) [43]	1.25	2.04	3.02	5.61
	Cu(II) [50]	3.72	4.55	6.53	-
	Zn(II) [42]	2.95	5.60	6.30	-
	Cd(II) [42]	2.20	3.93	5.11	5.81
	Ni(II) [71]	1.04	1.51	2.32	3.05
1-octyl-4-methylimidazole (**12**)	Co(II) [43]	1.34	2.14	3.28	5.79
	Cu(II) [50]	3.85	4.49	6.57	-
	Zn(II) [42]	2.04	3.50	6.20	6.90
	Cd(II) [37]	1.26	2.20	2.93	3.91
	Ni(II) [37]	0.69	1.04	2.00	2.92
1-decyl-4-methylimidazole (**13**)	Co(II) [43]	1.40	2.95	3.60	5.83
	Cu(II) [50]	3.94	4.53	6.60	-
	Zn(II) [42]	4.30	6.90	7.70	12.50
	Cd(II) [65]	1.45	2.25	2.48	3.14
	Ni(II) [65]	0.55	0.86	1.48	-

**Table 5:** The stability constants (log β_n_) of Cd(II), Ni(II), and Zn(II) complexes with 1-alkyl-2,4-dimethylimidazoles in aqueous solutions at 25 °C, ionic strength 0.5 mol/dm^3^ (KNO_3_).

Carrier	Metal Ion	log β_1_	log β_2_	log β_3_	log β_4_
1-octyl-2,4-dimethylimidazole (**14**) [61]	Zn(II)	1.65	2.17	4.48	6.39
	Cd(II)	1.17	2.53	4.21	5.68
	Ni(II)	0.09	0.22	1.11	2.05
1-decyl-2,4-dimethylimidazole (**15**) [69]	Zn(II)	2.17	4.48	6.39	9.87
	Cd(II)	2.53	4.21	5.68	7.56

**Table 6:** An example of the transport of Cu(II) ions across PIMs doped with alkylimidazole derivatives from chloride or nitrate solutions reported in the literature.

Carriers		Target Solutions	Solution	Cu(II) Initial Flux, J_0_, 10^−6^ mol/m^2^s	Ref.
1-alkylimidazole	**1**–**5**	Cu–Zn–Co–Ni	chloride	4.28–6.36	[59]
	**1**–**5**	Cu–Zn–Co–Ni	nitrate	5.16–7.03	[57]
	**2**	Cu–Zn–Co–Ni	nitrate	3.86	[58]
1-alkyl-2-methylimidazole	**6**, **8**, **9**	Cu–Zn–Co–Ni	nitrate	1.98–2.42	[63]
	**6**	Cu–Zn–Co–Ni	nitrate	1.15	[64]
	**7**	Cu–Zn–Co–Ni	nitrate	2.27	[58]
	**9**	Cu–Zn–Co–Ni	nitrate	2.35	[65]
1-alkyl-4-methylimidazole	**11**	Cu–Zn–Co–Ni	nitrate	2.43	[58]

**Table 7:** The copper(II) separation coefficients in relation to Zn(II), Co(II), and Ni(II) from 4-component Cu–Zn–Co–Ni equimolar solutions after a 24-h transport.

Separation Coefficients Cu(II)/M(II)											
Nitrate Solutions						Chloride Solutions					
Carrier		Zn	Co	Ni	Ref.	Carrier		Zn	Co	Ni	Ref.
1-alkylimidazole	**1**	4.3	39.7	46.9	[57]	1-alkyl-imidazole	**1**	3.7	17.8	35.7	[59]
	**2**	4.3	30.4	39.1	[57]		**2**	3.6	9.0	26.4	[59]
	**3**	3.7	22.6	33.2	[57]		**3**	3.2	21.6	24.8	[59]
	**4**	3.6	21.9	29.2	[57]		**4**	3.1	29.8	34.5	[59]
	**5**	3.5	22.0	22.7	[57]		**5**	3.0	14.8	15.5	[59]
1-alkyl-2-methylimidazole	**6**	3.9	24.8	59.1	[63]						
	**7**	3.1	15.1	35.1	[58]						
	**8**	4.0	14.5	36.3	[63]						
	**9**	2.8	12.1	38.0	[63]						
1-alkyl-4-methylimidazole	**11**	2.8	11.6	32.8	[58]						

**Table 8:** The values of Cu(II) recovery factors (RF) after a 24-h transport across PIMs doped with alkylimidazoles.

	Alkylimidazole						
Carrier	1	2	6	7	8	9	11
RF, %	99.4	98.0	83.2	95.0	86.5	94.5	93.0
Ref.	[57]	[57]	[63]	[58]	[63]	[63]	[58]

**Table 9:** An example of the transport of Zn(II) ions across PIMs doped with alkylimidazole derivatives from chloride, nitrate or sulphate solutions reported in the literature.

Carriers		Target Solutions	Solution	Zn(II) Initial Flux, J_0_, 10^−6^ mol/m^2^s	Ref.
1-alkylimidazole	**1**–**5**	Co–Ni–Zn	nitrate	1.79–2.50	[60]
	**1**–**5**	Zn–Mn	sulphate	1.97–2.65	[62]
	**3**	Zn–Cd–Ni	nitrate	10.76	[61]
1-alkyl-2-methylimidazole	**8**	Zn–Cd–Ni	nitrate	8.49	[61]
	**9**	Zn–Cd	nitrate	-	[69]
1-alkyl-4-methylimidazole	**10**	Zn–Cd–Co–Ni	chloride	3.64	[67]
	**10**	Zn–Cd	chloride	3.85	[67]
	**10**	Zn–Cd–Co	chloride	3.73	[67]
	**12**	Zn–Cd–Ni	nitrate	8.97	[61]
	**12**	Zn from waste	chloride	-	[68]
	**13**	Zn–Cd–Co–Ni	chloride	3.72	[66]
	**13**	Zn–Co	chloride	4.10	[66]
	**13**	Zn–Cd	chloride	3.89	[66]
	**13**	Zn–Ni	chloride	4.25	[66]
	**13**	Zn–Cd	nitrate	8.79	[69]
1-alkilo-2,4-dimethylimidazole	**14**	Zn–Cd–Ni	nitrate	25.44	[61]
	**15**	Cd–Zn	nitrate	25.44	[69]

**Table 10:** Zinc(II) separation coefficients compared to Cd(II), Co(II) and Ni(II) from 4-component Zn–Cd–Co–Ni equimolar solutions after a 24-h transport.

Separation Coefficients S_Zn(II)/M(II)_					
Chloride Solutions					
Carrier		Cd(II)	Co(II)	Ni(II)	Ref.
1-alkyl-4-methylimidazole	**10**	12.9	23.4	40.8	[67]
	**13**	11.6	24.8	33.8	[66]

**Table 11:** Zinc(II) separation coefficients from 3-component Zn–Co–Ni equimolar solutions after a 24-h transport across PIMs doped with alkylimidazole.

Separation Coefficients Zn(II)/M(II)					
Carrier		Solution	Co	Ni	Ref.
1-alkylimidazole	**1**	nitrate	9.4	11.9	[60]
	**2**		7.4	9.3	[60]
	**3**		6.9	8.1	[60]
	**4**		6.7	7.0	[60]
	**5**		6.4	7.8	[60]
1-alkyl-4-methylimidazole	**10**	chloride	9.8	24.9	[67]

**Table 12:** Zinc(II) separation coefficients from 2-component equimolar solutions after a 24-h transport.

Separation Coefficients Zn(II)/M(II)				
Carrier	Mixture	Solution	S_Zn(II)/M(II)_	Ref.
**1**	Zn–Mn	sulphate	19.7	[62]
**2**	Zn–Mn	sulphate	15.7	[62]
**3**	Zn–Mn	sulphate	14.2	[62]
**4**	Zn–Mn	sulphate	12.8	[62]
**5**	Zn–Mn	sulphate	11.0	[62]
**13**	Zn–Cd	chloride	7.9	[66]
**10**	Zn–Cd	chloride	9.4	[67]
**9**	Zn–Cd	nitrate	10.8	[69]
**13**	Zn–Cd	nitrate	9.9	[69]
**15**	Zn–Cd	nitrate	24.7	[69]
**13**	Zn–Co	chloride	27.3	[66]
**13**	Zn–Ni	chloride	22.4	[66]
**12**	Zn–Ni	chloride	30.6	[68]
**12**	Zn–Ni	sulphate	29.9	[68]

**Table 13:** The recovery factor (RF) of zinc during the transport of a mixture of ions across PIMs doped with alkylimidazoles after a 24-h transport.

**Mixture**	**Zn–Co**		**Zn–Mn**					**Zn–Cd**		
Carrier	**1**	**5**	**1**	**2**	**3**	**4**	**5**	**9**	**13**	**15**
RF, %	89	96	83	85	87	90	92.5	87	83	94
Ref.	[60]		[62]					[69]		
**Mixture**	**Zn–Co–Ni**		**Zn–Cd–Ni**				**Zn–Cd–Co–Ni**			
Carrier	**1**	**5**	**3**	**8**	**12**	**14**	**5**			
RF, %	76	87	87	92	94	95.5	96.9			
Ref.	[60]		[61]				[66]			

**Table 14:** Surface parameters of polymeric membranes with alkyl derivatives as carriers.

Carrier	Effective Pore Size, µm	Tortuosity (τ)	Roughness (R_q_), nm	Porosity, %	Ref.
**1**	-	2.42	3.9	24.2	[59]
**1**	0.050	2.32	5.9	-	[62]
**2**	-	-	3.9	21.1	[58]
**2**	0.053	2.35	6.1	-	[62]
**3**	0.054	2.38	6.5	-	[62]
**4**	0.055	2.64	6.7	-	[62]
**5**	0.057	2.81	7.2	18.1	[69]
**6**	0.060	2.83	2.2	16.0	[64]
**7**	-	-	2.4	16.3	[58]
**8**	0.054	2.38	6.1	-	[61]
**9**	0.060	2.85	6.7	16.0	[69]
**10**	0.061	2.83	6.6	15.8	[67]
**11**	-	-	2.7	18.9	[58]
**12**	0.058	2.75	6.5	-	[61]
**13**	0.060	2.85	6.7	-	[66]
**14**	0.062	2.60	6.0	-	[61]
**15**	0.065	2.45	5.8	23.7	[69]

**Table 15:** Degradation temperatures and weight losses of CTA-*o*-NPPE membranes with alkylimidazoles.

Carrier	The First Step		The Second Step		Ref.
	Temp. °C	Weight Loss, %	Temp. °C	Weight Loss, %	
**1**	211.3	74.63	360.7	13.90	[57,60]
**5**	227.7	61.30	358.6	18.90	[60]
**9**	251.3	80.09	359.1	5.12	[65]
**10**	220.1	80.57	327.0	5.88	[67]
**12**	230.0	80.00	370.0	15.00	[68]
**13**	230.3	79.14	342.4	7.46	[66]

**Table 16:** Diffusion coefficients for the transport of non-ferrous metal ions across PIMs with alkylimidazoles.

Carrier	Cations	Permeability (P), m/s	Diffusion Coefficients D_0_, cm^2^/s	Normalized Diffusion Coefficients D_0,n_, cm^2^/s	Ref.
**1**	Cu(II)	4.28 × 10^−3^	3.44 × 10^−11^	4.17 × 10^−10^	[59]
	Zn(II)	1.17 × 10^−3^	2.18 × 10^−11^	2.64 × 10^−10^	
	Co(II)	0.24 × 10^−3^	5.17 × 10^−12^	6.26 × 10^−11^	
	Ni(II)	0.12 × 10^−3^	3.03 × 10^−12^	3.67 × 10^−11^	
**6**	Cu(II)	3.86 × 10^−3^	3.02 × 10^−11^	1.75 × 10^−12^	[63]
	Zn(II)	1.10 × 10^−3^	1.94 × 10^−11^	1.12 × 10^−12^	
	Co(II)	4.04 × 10^−4^	1.01 × 10^−11^	5.86 × 10^−13^	
	Ni(II)	3.08 × 10^−4^	7.70 × 10^−12^	4.43 × 10^−13^	
**3**	Zn(II)	-	2.04 × 10^−8^	2.11 × 10^−9^	[61]
	Cd(II)	-	7.16 × 10^−9^	7.40 × 10^−10^	
	Ni(II)	-	4.39 × 10^−11^	4.53 × 10^−12^	
**8**	Zn(II)	-	1.92 × 10^−8^	2.01 × 10^−9^	[61]
	Cd(II)	-	6.76 × 10^−9^	7.14 × 10^−10^	
	Ni(II)	-	4.15 × 10^−11^	4.27 × 10^−12^	
**12**	Zn(II)	-	1.72 × 10^−8^	1.98 × 10^−9^	[61]
	Cd(II)	-	6.34 × 10^−9^	6.75 × 10^−10^	
	Ni(II)		4.59 × 10^−11^	4.83 × 10^−12^	
**14**	Zn(II)	-	1.53 × 10^−8^	1.74 × 10^−9^	[61]
	Cd(II)	-	6.06 × 10^−9^	6.58 × 10^−10^	
	Ni(II)	-	4.72 × 10^−11^	4.91 × 10^−12^	
**10**	Zn(II)	-	6.94 × 10^−9^	3.85 × 10^−10^	[67]
	Cd(II)	-	1.56 × 10^−10^	8.66 × 10^−12^	
	Co(II)	-	8.16 × 10^−11^	4.28 × 10^−12^	
	Ni(II)	-	7.71 × 10^−11^	4.53 × 10^−12^	
